# Diagnostic accuracy of contrast-enhanced ultrasound for detecting bland thrombus from inferior vena cava tumor thrombus in patients with renal cell carcinoma

**DOI:** 10.1590/S1677-5538.IBJU.2019.0304

**Published:** 2020-01-13

**Authors:** Qiuyang Li, Zhongxin Wang, Xin Ma, Jie Tang, Yukun Luo

**Affiliations:** 1 Department of Ultrasound, Chinese PLA General Hospital, Beijing 100853, China;; 2 Department of Urology, Chinese PLA General Hospital, Beijing 100853, China

**Keywords:** Carcinoma, Diagnosis, Thrombosis

## Abstract

**Purpose:**

To evaluate the role of contrast-enhanced ultrasound (CEUS) in differentiating bland thrombus from tumor thrombus of the inferior vena cava (IVC) in patients with renal cell carcinoma (RCC).

**Materials and Methods:**

We retrospectively investigated 30 consecutive patients who underwent robot-assisted radical nephrectomy with IVC thrombectomy and had pathologically confirmed RCC. All patients underwent US and CEUS examination. Two off-line readers observed and recorded thrombus imaging information and enhancement patterns. Sensitivity, specificity, accuracy, positive predictive value and negative predictive value for bland thrombus were assessed.

**Results:**

Of the 30 patients, no adverse events occurred during administration of the contrast agent. Early enhancement of the mass within the IVC lumen on CEUS was an indicator of tumor thrombus. Bland thrombus showed no intraluminal flow on CEUS. There were eight (26.7%) patients with bland thrombus, including three level II, two level III, and three level IV. There were three cases with cephalic bland thrombus and five cases with caudal bland thrombus. Three caudal bland thrombi extended to the iliac vein and underwent surgical IVC interruption. Based on no intraluminal flow, for bland thrombus, CEUS had 87.5% sensitivity, 100% specificity, 96.7% accuracy, 100% positive predictive value and 95.6% negative predictive value.

**Conclusion:**

Our study demonstrates the potential of CEUS in the differentiation of bland and tumor thrombus of the IVC in patients with RCC. Since CEUS is an effective, inexpensive, and non-invasive method, it could be a reliable tool in the evaluation of IVC thrombus in patients with RCC.

## INTRODUCTION

Approximately 4-10% of cases of renal cell carcinoma (RCC) are associated with inferior vena cava (IVC) tumor thrombus (1, 2). Embolization of tumor and bland thrombus is a potential fatal complication for patients undergoing radical nephrectomy and tumor thrombectomy (3). Several surgical strategies have been proposed to prevent the dissemination of bland thrombus after surgery, including placement of a filter in the IVC, and IVC ligation and segmental resection (4). Bland thrombus is associated with adverse survival outcome in patients treated surgically for RCC with IVC tumor thrombus (5). Therefore, discrimination of bland and tumor thrombus is of clinical significance for determining the therapeutic approach and predicting survival.

Contrast-enhanced ultrasound (CEUS) is emerging as a valuable imaging modality that complements and enhances conventional vascular US imaging in clinical and scientific settings. US contrast agents are gas-filled microbubbles that are injected into the bloodstream and serve as strict intravascular reflectors of ultrasound waves, providing real-time assessment of the dynamic temporal and spatial heterogeneity of the macro––and micro-vascular perfusion (6). Contrast-specific image processing techniques based on the nonlinear scattering properties from microbubbles allow enhancement of vascular structures and quantification of tissue perfusion. Therefore, CEUS is effective in evaluating tissue vascularity and has been widely used in different organs, lesions and vascular diseases in recent years (7–11). Previous reports have concluded that CEUS is an excellent method for differentiation of malignant from benign portal vein thrombosis in hepatocellular carcinoma (12–14).

To the best of our knowledge, the value of CEUS has not been studied for differentiation between bland and tumor thrombus of the IVC in patients with RCC. The purpose of this study was to investigate the value of CEUS imaging in distinguishing bland thrombus from tumor thrombus in the IVC in patients with RCC.

## MATERIALS AND METHODS

### Patients

This study was approved by the Ethics Committee of the PLA General Hospital, China and written informed consent was obtained from each patient. The study included 30 consecutive patients who underwent robot-assisted radical nephrectomy with IVC thrombectomy and had pathologically confirmed RCC between October 2017 and August 2018. IVC thrombus level was categorized as previously described (1). Patients with level 0 thrombus who did not undergo IVC resection were excluded from the study. A total of 30 patients with level I-IV thrombus formed the analytical cohort for this study. Patient characteristics (age, gender, body mass index, clinical stage, thrombus classification, and thrombus length) were collected and analyzed (Table-1).

**Table 1:** Descriptive clinicopathologic characteristics of the 30 patients with clear cell renal cell carcinoma and inferior vena cava tumor thrombus.

Characteristics		Results
**Patients, n**		30
**Median age, yr (interquartile range)**		57.6 (46.5-65.3)
**Male/Female (n)**		20/10
**Mean body mass index, kg/m^2^ (range)**		24.6 (17.8-30.5)
**Affected kidney (n)**		
	Left	9
	Right	21
**Mean tumor size, cm (range)**		7.5 (3.1-15.7)
**Clinical stage (n)**		
	T3aN0M0	3
	T3bN0M0	18
	T3bN0M1	5
	T3cN0M1	2
	T3bN0M1	2
**IVC thrombus classification (n)**		
	Level I	10
	Level II	14
	Level III	3
	Level IV	3
**Mean IVC thrombus length cm (range)**		7.7 (4.8-13.6)
**Presence of bland thrombus (n)**		8
**Superior bland thrombus (n)**		3
**Caudal bland thrombus (n)**		5
**Surgical strategy during IVC thrombectomy**		
**Incision of the IVC for thrombectomy (n)**		25
**IVC segmental transection(n)**		5

### US contrast agents (UCAs)

UCAs are gas-containing microspheres with an outer shell of lipid, protein or polymer (15). With a diameter ranging from 1 to 10μm, these micro-bubbles are roughly the size of a red blood cell. This size allows them to pass through capillaries and be delivered to any tissue that maintains circulation, while avoiding extravascular passage (16, 17). In practice, UCAs cause a low incidence of adverse effects and are considered safe for patients with decreased renal function. These patients benefit from the fact that the UCAs are not excreted into the urine and therefore not nephrotoxic (18). UCAs are also used in the pediatric population, and in numerous other documented areas. The US Food and Drug Administration recently approved the use of Lumason^TM^ (marketed as SonoVue^TM^, Bracco, Milan, outside the US) for pediatric liver imaging (19).

### CEUS data acquisition

Patients were advised to follow a low-residual diet the day before CEUS examination and to fast in the morning of the day of the examination. US and CEUS were performed by the same sonographer with 5 year's experiences with abdominal CEUS. Examinations were performed transabdominally with the patients in different positions. Both US and CEUS were performed with a Resona 7 (Mindary Medical Solutions, Shenzhen, China) equipped with UWN+software using an SC6-1U abdominal transducer, pulse inversion (PI) and power modulation (PM) modes at a mechanical index of 0.08. Contrast agent SonoVue^®^ (Bracco, Milan, Italy) was used for CEUS. SonoVue^®^ is a second––generation sulfur hexafluoride microbubble contrast agent that provides strong and continuous real-time imaging. A 1.5mL contrast agent bolus was injected through a 20-gauge cannula followed by 5mL normal saline flushing, using a three-way stopcock to ensure that no residual contrast agent was left in the intravenous catheter. Images and cine clips of the entire CEUS examination were stored digitally for offline analysis.

### CEUS image analysis

All patients underwent US, which revealed thrombus height, length, width, boundaries, modalities, echo features and color Doppler flow imaging information. After CEUS, two off-line readers observed and recorded thrombus enhancement patterns. Both readers were skilled in urological sonography with >5 years of CEUS examination experience, and were blinded to patient final diagnoses and clinical and radiological information. If there was disagreement between the two readers, another pair of senior physicians re-evaluated the clips until a final conclusion was reached. Evaluation of the CEUS findings was conducted as follows: early enhancement of a mass within the IVC lumen on CEUS was an indicator of tumor thrombus, and IVC bland thrombus showed no intraluminal flow on CEUS.

### Histological examination

Within 7 days after CEUS examination, all patients underwent robot-assisted radical nephrectomy with IVC thrombectomy. Histological diagnosis was performed according to the World Health Organization Classification of Tumors of the Urinary System and Male Genital Organs (20). Presence of bland thrombus was defined as any noted bland thrombus within the operative report or noted in the pathology report.

### Postoperative treatment and follow-up

For follow-up and surveillance of the patients, US, computed tomography (CT) and magnetic resonance imaging (MRI) scans of the chest, abdomen and pelvis were performed every 6 months.

## RESULTS

Technical success for CEUS was obtained in all the patients. Every CEUS examination was of sufficient quality to enable analysis and no relevant motion artefacts were encountered. No adverse events occurred during administration of the contrast agent. The information from surgery and pathology confirmed the diagnosis of clear cell RCC in all the 30 patients who underwent nephrectomy with IVC thrombectomy. The thrombus level was I in 10 patients (33.3%), II in 14 patients (46.7%), III in three patients (10%), and IV in three patients (10%).

We used robotic techniques that depend on the level of venous thrombus, as described previously and summarized by our department (21–23). For a thrombus inferior to the first porta hepatis (level I and part of level II), we ligated some short hepatic veins. For a thrombus between the first porta hepatis and second porta hepatis (level II), we mobilized the right lobe of the liver from the IVC by ligating additional short hepatic veins. For a thrombus near or above the second porta hepatis but below the diaphragm (level III), we mobilized the right and left lobes of the liver to obtain high proximal control of the suprahepatic and infradiaphragmatic IVC, and simultaneously clamped the first porta hepatis. For a thrombus above the diaphragm and in the right atrium (level IV), we established cardiopulmonary bypass (CPB), and performed the thoracoscopy-assisted thrombectomy for the intra-atrial part of the thrombus under CPB. The infradiaphragmatic part was treated in a manner similar to that of level III.

Cephalic bland thrombus and short caudal bland thrombus were treated as tumor thrombus. For patients with long caudal bland thrombus associated with tumor thrombus, in which the thrombus filled the IVC lumen and where there was excellent collateral circulation, we simply ligated the IVC above and below the thrombus, and the renal vein using an Endo GIA stapler (Medtronic, Minneapolis, MN, USA) with a 45mm vascular load.

**Figure 1:**
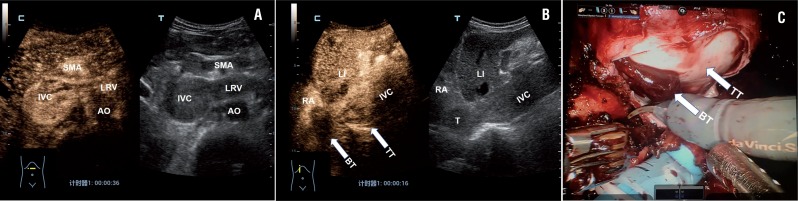
Example case of left-sided renal tumor with a level IV IVC thrombus in a 55 years old male patient. A) CEUS scan obtained 36 s after injection of microbubbles showed heterogeneous enhancement within the thrombus involving the LRV and IVC. In situ mapping of blood flow using B mode US image of thrombus (right) with CEUS mode (left). B) CEUS scan showed no enhancement within the bland thrombus of the superior IVC in RA (arrows) and strong enhancement within the tumor thrombus of the IVC (arrows). C) Intraoperative robotic view of the bland thrombus of the superior IVC (arrow). LRV=Left renal vein, AO=Abdominal aorta, BT=bland thrombus, LI=liver, SMA=superior mesenteric artery, T=thrombus, TT=tumor thrombus, RA=right atrium.

**Figure 2:**
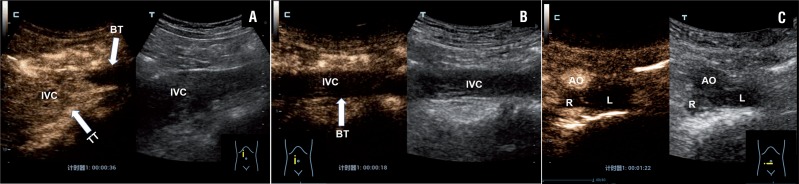
Example case of right-sided renal tumor with a level III IVC thrombus in a 60-year-old male patient. A) CEUS scan showed strong enhancement within the tumor thrombus of the proximal segment of the IVC (arrows) and no enhancement within the bland thrombus of the distal segment (arrows). B) CEUS scan showed no enhancement within the bland thrombus of the distal segment of the IVC (arrows). C) CEUS scan showed no enhancement within the bland thrombus of the bilateral iliac vein.

**Figure 3:**
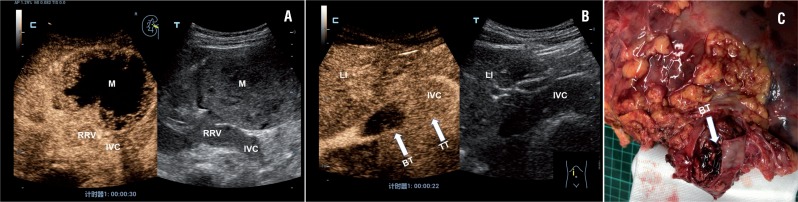
Example case of right-sided renal tumor with a level II IVC thrombus in a 45-year-old female patient. A) B-mode imaging showed a mass at the mid and lower posterolateral right kidney, solid hypoechoic thrombus in RRV and IVC (right). CEUS scan showed heterogeneous enhancement in the right renal mass and homogeneous, complete enhancement of the tumor thrombus in the RRV and IVC (left). B) CEUS scan showed strong enhancement within the tumor thrombus of the distal segment of the IVC (arrows) and no enhancement within the bland thrombus of the proximal segment of the IVC (arrows). C) Postoperative gross specimens showed tumor thrombus with superior bland thrombus in the IVC.

There were eight (26.7%) patients with bland thrombus, including three level II, two level III, and three level IV. There were three patients with cephalic bland thrombus (Figure-1) and five with caudal bland thrombus. Three caudal bland thrombi extended to the iliac vein (Figure-2). Enhancement pattern of the thrombi helped to distinguish bland from tumor thrombi (Figure-3). Based on 100% agreement between the two observers, there was 87.5% (7/8) agreement between CEUS and intraoperative findings in differentiating bland from tumor thrombi. Diagnosis of one case of tiny caudal bland thrombus was missed by CEUS. Based on no intraluminal flow, for bland thrombus, CEUS had 87.5% sensitivity, 100% specificity, 96.7% accuracy, 100% positive predictive value and 95.6% negative predictive value.

Five patients had the IVC interrupted by ligation below the tumor thrombus. After thrombectomy, no intraoperative IVC filter placement was performed through the cavatomy, and none of the 30 patients had a bland thrombus pulmonary embolus during or after surgery. Eight patients developed mild to severe renal dysfunction. We isolated and excised lymph nodes proximal to the IVC in 12 patients, and positive findings were observed in four cases. During the follow-up of a median of 12 months (range 10-20 months), no tumor embolus infringement of the IVC wall or positive lymph nodes or distant metastasis was found.

## DISCUSSION

The safety and feasibility of robot-assisted laparoscopic IVC thrombectomy have been investigated in our previous studies (21–23). We found that coexisting bland thrombus was not uncommon in RCC patients with IVC tumor thrombus. In the present study, we found eight (26.7%) patients with coexistent bland thrombus, the incidence was higher than that reported in the literature (2, 4, 5). There could be two reasons for this. First, only a small number of patients were included in our study. Second, the patients admitted to our center have complex conditions. Bland thrombus only occurred in patients with level II-IV tumor thrombus. The presence of bland thrombus associated with tumor thrombus should alert the surgical team to a possible complex and challenging surgical encounter. The surgical significance of coexisting bland thrombus has been reported (4). In our study, in three patients with cephalic bland thrombus, we suggested immediate surgery in case they progressed rapidly. For the surgical strategy, we regard the level of bland thrombus as tumor thrombus and occlude the IVC superior to the bland thrombus. Small distal bland thrombus can be removed directly, whereas most distal thrombi extend to the iliac bifurcation and cannot be removed. In the latter cases, we aim for negative margins by identifying the distal margin of the tumor thrombus and then proceeding to ligate and divide the IVC. Moreover, we perform segmental resection of the IVC when the tumor thrombus is adherent to the vessel wall or if there is no identifiable interface between tumor and bland thrombi (22, 23). Association of bland with tumor thrombus should alert the surgical team to a potentially challenging surgical situation. About half of the patients with bland thrombus require IVC ligation or segmental resection (4, 5). Precise preoperative imaging to differentiate bland from tumor thrombus is a key step in achieving the surgical goal with minimal morbidity.

Although conventional venography remains the “gold standard” for diagnosing vein thrombosis, it is invasive and exposes patients to ionizing radiation. In clinical practice, to reliably differentiate bland from tumor thrombus, CT and MRI rely on the use of contrast media. Apart from allergic reactions, CT contrast media are associated with an increased risk of renal failure and MRI contrast agents carry a risk of nephrogenic systemic fibrosis in patients with highly impaired renal function (24, 25). RCC patients with bland or tumor thrombus in the IVC are at an especially high risk for nephrogenic systemic fibrosis, as they often suffer from impaired renal function (26). Furthermore, with recent literature reporting gadolinium deposition in the brain and other body tissues of unknown clinical significance after repeated administration of gadolinium-based contrast agents, concerns for patient safety are rising and institutional review of policies for gadolinium administration is warranted (27). UCAs are administered safely in various applications with minimal risk to patients. They are not excreted through the kidneys, and can be safely be administered to patients with renal insufficiency with no risk of contrast-related nephropathy or nephrogenic systemic fibrosis. UCAs have a low rate of anaphylactoid reactions (1:7000 patients, 0.014%), significantly lower than the rate with iodinated state-of-the-art CT agents (35-95:100.000 patients, 0.035-0.095%), but comparable to the rate of severe anaphylactoid reactions associated with gadolinium-based contrast agents at 0.001-0.01% (10).

CEUS provides real-time examination of tissue enhancement. Arterial neovascularization within a tumor thrombus results in arterial enhancement, which, on real-time imaging, can be easily distinguished from venous enhancement by its timing and intermittent pulsation. CEUS has a high intrinsic sensitivity because the microbubbles produce echoes that are thousand billion times stronger than the echo from similar-sized red blood cells. This, together with the background tissue suppression using pulse inversion methods, results in high intrinsic contrast between contrast-enhanced blood and tissue (14, 28). Previous investigators have shown promising results with CEUS for discriminating malignant or benign venous thrombus in liver (29). These results were confirmed and extended in a subsequent study on a large series of patients with hepatic cirrhosis in which CEUS showed a high sensitivity (94%) and specificity (96%) in differentiating malignant versus bland portal vein thrombosis. Based on all these data, the European Federation of Societies for Ultrasound in Medicine and Biology (EFSUMB) included the “differential diagnosis between malignant and benign portal vein thrombosis” among indications for CEUS in their updated guidelines (30).

**Figure 4:**
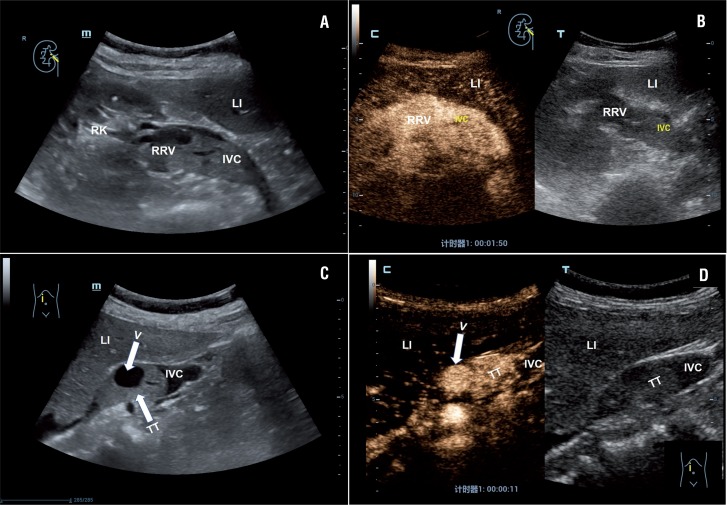
Example case of right-sided renal tumor with a level II IVC thrombus in a 53-year-old female patient. A) B-mode imaging showed solid hypoechoic thrombus in the RRV and IVC. B) CEUS scan showed more lasting, homogeneous, complete marked enhancement of the tumor thrombus in the RRV and IVC. There was adjacent liver washout at 1 min 50s post-injection. C) B-mode imaging showed a formed vessel in the tumor thrombus of the IVC (arrows). D) CEUS scan showed rapid and strong enhancement of the formed vessel in tumor thrombus 11s after injection of microbubbles.

Our study focused on identifying the individual CEUS features most helpful in differentiating bland and tumor thrombi, and showed a high sensitivity (87.5%) and specificity (100%) of CEUS. With regard to specific features, enhancement of the thrombus was the most important finding to diagnose tumor thrombus, with excellent interobserver agreement. Presence of formed vessels is another useful feature, which is detected more accurately with contrast enhancement due to the blood pool nature of the contrast agent (Figure-4). The tiny vessels in tumor thrombus can be seen on CEUS, but may be beyond the resolution threshold of conventional color Doppler ultrasound and therefore can be missed.

To the best of our knowledge, the present study is one of the first to investigate the utility of CEUS in distinguishing bland from tumor thrombus in IVC in patients with RCC.

There were two limitations to the present study. First, the retrospective single institution design and the small number of patients may have led to selection bias. Second, we did not compare CT and MRI findings due to the retrospective nature of the study and the comparison was not the purpose of this study.

## CONCLUSION

CEUS has high diagnostic accuracy for the differentiation of bland from tumor thrombi of the IVC in patients with RCC. Since CEUS is an effective, inexpensive, and non-invasive method, it could be a reliable tool for evaluation of thrombus in the IVC in patients with RCC.
